# Circulatory extracellular vesicle derived miR-195-5p promotes cellular apoptosis and suppresses cell proliferation in the buffalo endometrial primary cell culture

**DOI:** 10.1038/s41598-023-43530-y

**Published:** 2023-10-04

**Authors:** Ankit Pal, Seema Karanwal, Jatinder Singh Chera, Vipul Batra, Arumugam Kumaresan, Parul Sarwalia, Tirtha K. Datta, Rakesh Kumar

**Affiliations:** 1 Animal Genomics Laboratory, Animal Biotechnology Centre, National Dairy Research Institute, Karnal, India; 2 Theriogenelogy Laboratory, SRS of National Dairy Research Institute, Bengaluru, India

**Keywords:** Biotechnology, Cell biology, Developmental biology, Molecular biology

## Abstract

In pregnant animals, communication between the mother and conceptus occurs via extracellular vesicles (EVs) that carry several biomolecules such as nucleic acids (miRNAs, mRNAs), proteins, and lipids. At the time of implantation, the endometrium undergoes several morphological and physiological changes, such as angiogenesis, apoptosis, and cell proliferation regulation at the implantation site, to attain a receptive state. This study was conducted to detect pregnancy-specific miRNAs derived from extracellular vesicles in the systemic circulation of *Bubalus bubalis* (water buffalo) and to assess their functional significance in the modulation of endometrial primary cells. The extracellular vesicles were isolated from the blood plasma using a precipitation-based method and further characterized by various methods such as Differential light scattering, Nanoparticle tracking assay, Western blot, and transmission electron microscopy. The relative expression of the selected extracellular vesicles associated miRNAs (EV-miRNA) at different intervals (days 15, 19, 25, and 30) post artificial insemination (AI) was analyzed using RT-qPCR, and expression of miR-195-5p was found to be significantly higher (*P* < 0.01) in pregnant animals on day 19 post AI (implantation window) as compared to day 15 post AI. The elevated expression might indicate the involvement of this miRNA in the maternal-conceptus cross-talk occurring during the implantation period. The KEGG pathway enrichment and Gene Ontology analyses of the miR-195-5p target genes revealed that these were mostly involved in the PI3-Akt, MAPK, cell cycle, ubiquitin-mediated proteolysis, and mTOR signaling pathways, which are related to the regulation of cell proliferation. Transfecting the in vitro cultured cells with miR-195-5p mimic significantly suppressed (*P* < 0.05) the expression of its target genes such as *YWHAQ*, *CDC27, AKT-3, FGF-7, MAPK8, SGK1, VEGFA*, *CACAND1, CUL2, MKNK1,* and *CACAN2D1.* Furthermore, the downregulation of the miR-195-5p target genes was positively correlated with a significant increase in the apoptotic rate and a decrease in the proliferation. In conclusion, the current findings provide vital information on the presence of EV miR-195-5p in maternal circulation during the implantation window indicating its important role in the modulation of buffalo endometrium epithelial cells via promoting cell death. Altogether, the milieu of miR-195-5p may serve as a novel and potential molecular factor facilitating the implantation of the early embryo during the establishment of pregnancy in buffaloes. Thus, miR-195-5p may be identified as a unique circulatory EV biomarker related to establishing pregnancy in buffaloes as early as day 19 post-AI.

## Introduction

During pregnancy establishment, maternal-conceptus crosstalk provides essential signals for maternal recognition of pregnancy^1^. Successful pregnancy establishment depends on the exact timing of the maternal-conceptus crosstalk and synchronized transcriptional regulation between them. The placental cells release extracellular vesicles (EVs) that can cross the placental barrier between the mother and the conceptus to regulate various biological functions in the target cells^2^. EVs can enter physiological fluids such as plasma, urine, amniotic fluid, seminal plasma, milk, saliva, and uterine luminal fluid and then transmit their content to the target cells^3^. EVs secreted by the cells reflect the physiological state and function of the originating cells, such as the endometrium and primary trophoblast from the placenta^4–6^. EVs contain several biomolecules such as lipids, proteins, mRNA, and other small non-coding RNAs such as miRNAs^7^.

The miRNAs are a class of small (~ 22 nucleotides long) non-coding RNAs that negatively regulate gene expression by binding to the 3′—untranslated region of target mRNAs^8–11^ It has been observed that the abundance of miRNAs present within the EV differs in early pregnant, non-pregnant, and pregnancy loss^12^. The placental secreted miRNAs contribute to the circulating miRNA profile of the maternal blood and, thus, can be used as biomarkers for pregnancy detection^13^. During early pregnancy, i.e., the first trimester, the number of exosomes increases in the maternal circulation^14^. The association of miRNAs and exosomes increases the stability of these miRNAs, thereby preventing their degradation from RNase^15,16^. During pregnancy, the extracellular vesicles associated miRNA (EV-miRNA) profile follows specific trends in different trimesters, at term and pre-term birth, which indicates changes in maternal and conceptus tissue^17^.

The interaction between the uterus and the embryo is crucial for successful pregnancy development in mammals. During the implantation window, the endometrium attains a receptive state and undergoes several morphological and physiological changes, such as angiogenesis, apoptosis, and cell proliferation regulation at the implantation site^18–20^. Numerous studies have shown that silencing the essential miRNA processing enzymes causes developmental arrest or even embryonic death^21,22^. Placenta-derived miRNAs have been reported to be released into the maternal circulation after being packed into the EVs, thus providing intercellular communication between the mother and the conceptus^23^. Several studies have reported that the placenta-derived EV-miRNAs regulate the bi-directional interaction between endometrium and embryo at the time of implantation^24^. Placenta-derived EV-miRNAs present in the maternal circulation have been reported to protect the conceptus from the maternal immune response^25^. The functional roles of EV-miRNAs concerning pregnancy establishment have not been much explored. Therefore, to decipher the roles of EV-miRNAs in pregnancy establishment in water buffalo (*Bubalus bubalis*), the current study aims (i) To analyze the expression of pregnancy-specific EV-miRNAs present in the bloodstream of pregnant and non-pregnant buffaloes. (ii) Elucidating the role of pregnancy-associated EV-miRNA miR-195-5p, in the cell growth rate, proliferation, and apoptosis in the buffalo-cultured endometrial primary cells.

## Results

### Isolation and characterization of the EVs from blood plasma

**Figure 1:**
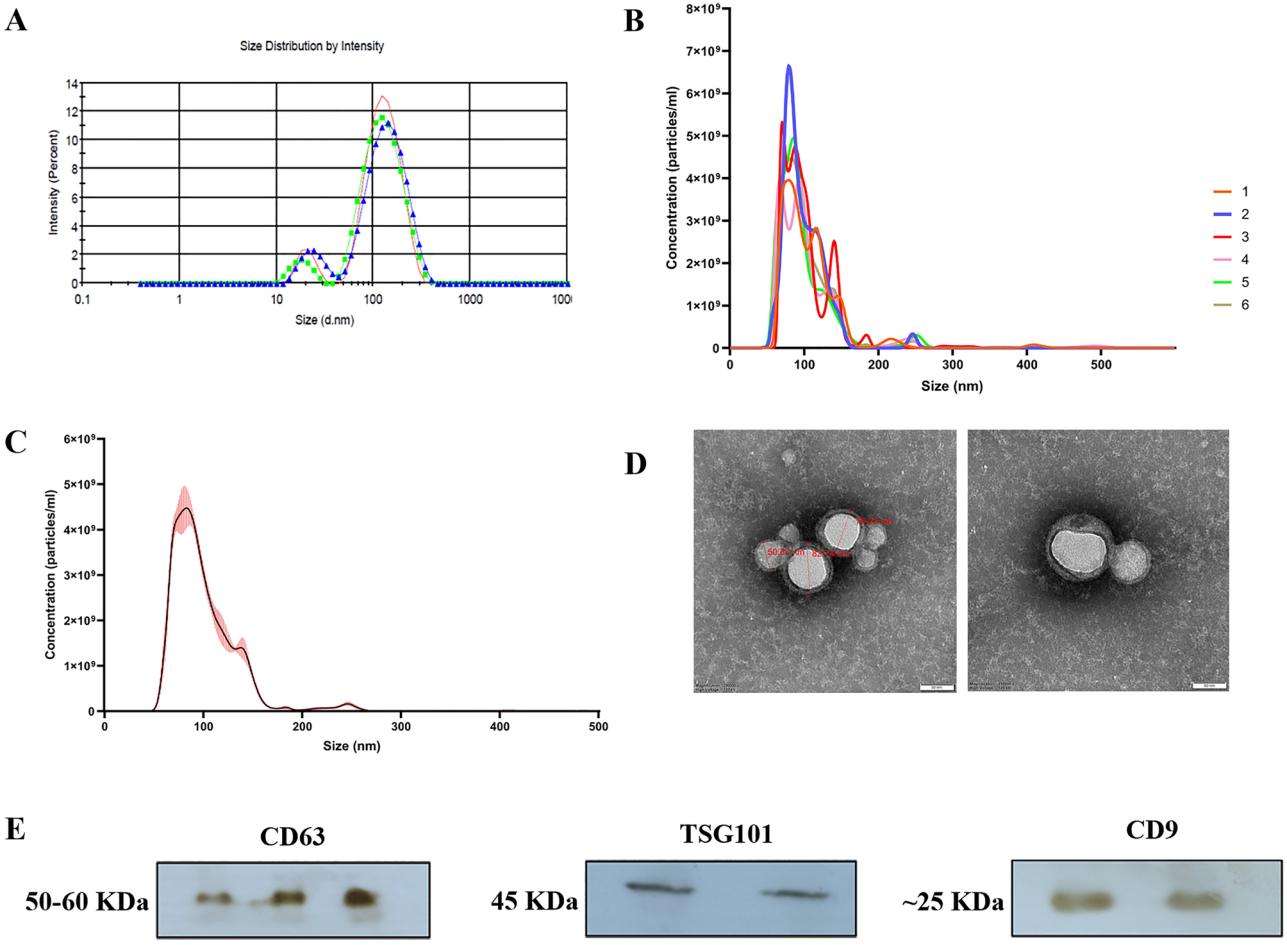
Characterization of extracellular vesicles. (**A**) Intensity-based Size distribution of buffalo blood plasma-derived EVs, as assessed by the Zetasizer nano zs particle sizer. Each curve shows means ± SD from three replicates in a representative experiment out of the three performed with similar results. (**B**) Nanoparticle Tracking Analysis (NTA) on the buffalo blood plasma-derived small EVs under 100× dilutions. FTLA size per concentration graph, taken as five replicates (**C**) graph represents the averaged FTLA size per concentration (particles/ml) (**D**) Transmission Electron Microscopy (TEM). TEM image of EVs derived from buffalo blood plasma. EVs were negatively stained with 1% phosphotungstic acid after removing the extra moisture. (Magnification-250000×, Scale bar—50 nm, 120 kV) (**E**) Identification of the CD9, CD63 and TSG101 EV-specific protein markers by the western blot analysis of isolated pooled EVs samples from the blood plasma of Murrah buffalo. Full blots are shown in Supplementary Fig. S3A–C.

Small EVs were isolated from the blood plasma of three randomly selected Murrah buffaloes using a precipitation-based method. These isolated EVs were then pooled and characterized further. The isolated EVs were characterized using Differential light scattering (DLS), Nanoparticle tracking assay (NTA), Transmission electron microscopy (TEM), and Western blot. The particle size obtained through the NTA and DLS was found to be mean size of 102 + /− 1.9 nm and Z-average size 91.3 nm, respectively (Fig. 1A–C), which lies within the expected size range of the small EVs, i.e., 30–150 nm^26^. The nanoparticle concentration was 2.55 × 10^11^ ± 7.09 × 10^9^ particles/ml in pooled blood plasma samples. Most of the nanoparticle concentration was found within the range of 40nm-150 nm, representing the small EV population. A video was obtained capturing the Brownian motion of the particles (Supplementary Video S1). General morphology and ultrastructure of blood plasma-derived EVs were assessed using transmission electron microscopy (TEM), allowing the visualization of round cup shape vesicles with lipid bilayer structures with diameters varying between 30 and 100 nm (Fig. 1 D). The presence of small EVs was also confirmed by Western blot using the EV protein markers, namely, a cluster of differentiation 63 (CD63), Tumor susceptibility gene101 (TSG101), a cluster of differentiation CD9, and EV negative marker Calnexin (Fig. 1E) (Supplementary Fig. S3A–D).

### Expression profile of the selected circulatory EV—miRNAs in pregnant and non-pregnant buffaloes post different days of insemination

**Figure 2:**
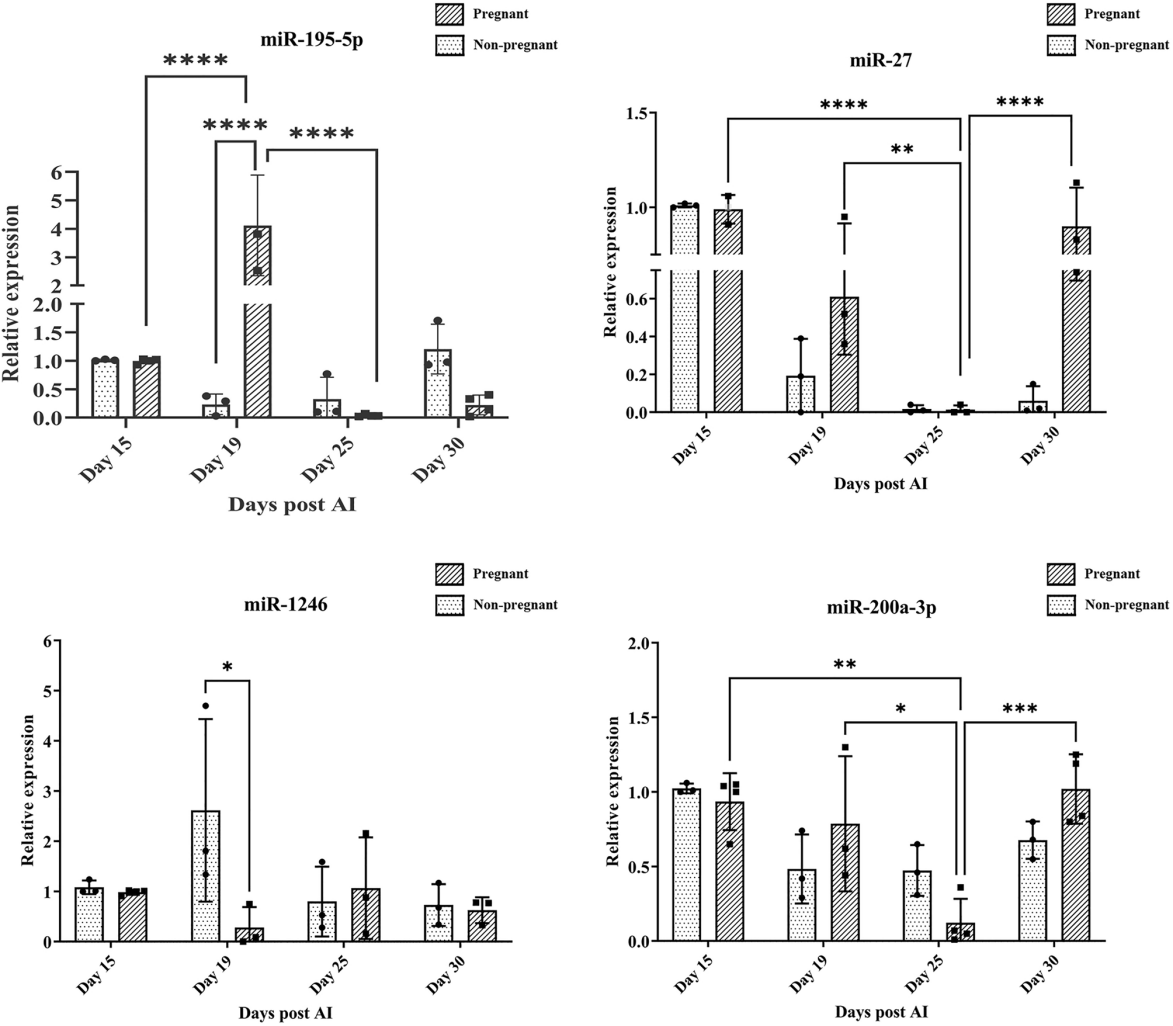
The pattern of expression of EV—miRNA in blood plasma. The Relative expression profiles of the EV-miRNA across the pregnant vs. non-pregnant buffalo on different days after insemination. Expression values were normalized to the mean of let-7 and miR-16a reference controls. Day 15 in pregnant and non-pregnant animals was chosen as the calibrator. Days post insemination is identified on the X-axis. (Pregnant animals (n = 3) and non-pregnant animals (n = 3) (*P < 0.05; **P < 0.01; and ****P < 0.0001).

The relative expression profiles of the selected panel of EV- miRNAs were generated using RT-qPCR at four different intervals post AI, namely, on days day 15, 19, 25, and 30, in three pregnant and three non-pregnant Murrah buffaloes. A dynamic expression of the candidate miRNAs on different days post-insemination was observed. The miR-195-5p exhibited a significant increase in the expression level from day 15 to 19 (*P* < 0.0001) post-AI, with a sharp decrease on day 25. Furthermore, the expression of miR-195-5p was much higher in the pregnant animals compared to the non-pregnant animals on day 19. The expression of EV-associated miR-200a-3p and miR-27 was observed to be decreased on day 19 and day 25 compared to day 15 in pregnant animals. The expression of miR-1246 was decreased on day 19 but later increased on day 25 of pregnancy. The expression of miR-27 and miR-200a-3p increased on day 30 in pregnant animals (Fig. 2). In bovine species, day 19–20 of pregnancy is referred to as the implantation window and trophoblast attachment begins during this phase. We wanted to explore the functional significance of increased expression of miRNA on day 19 of pregnancy.

### In-silico target gene prediction of miR-195-5p

**Figure 3:**
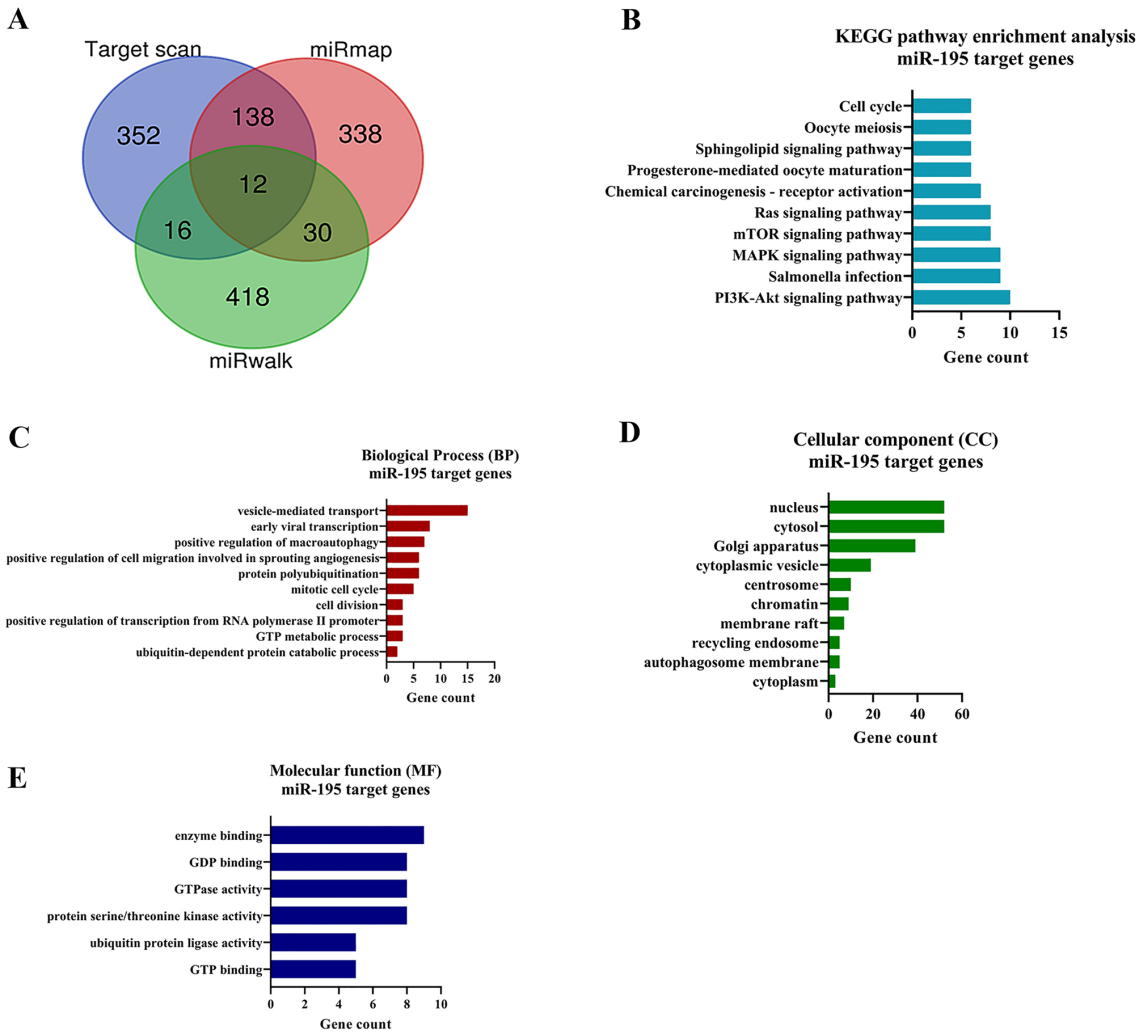
(**A**) miRNA target prediction. Venn diagram of the number of miR-195-5p targets predicted by each tool (**B**) KEGG pathway enrichment analysis. Kyoto Encyclopedia of Genes and Genomes pathway enrichment analysis of miR-195-5p (Top 10 highly gene enriched with significance (*P* < 0.05) target genes by Database for Annotation, Visualization and Integrated Discovery software. (Top 10). (**C**–**E**) Gene ontology. Gene ontology (GO) enrichment analysis for miR-195-5p targets in the category of biological processes. Gene ontology analysis of miRNA target genes according to biological process, cell component, and molecular function.

The potential target genes for miR-195-5p were determined by in silico analysis using different miRNA target prediction tools, namely miRWalk, TargetScan, and miRmap. The clustering analysis results of all predicted miRNA target genes are presented in Fig. 3A, Supplementary Table S3. It revealed a total of 196 target genes identified by a minimum of two databases after clustering through the Venn diagram (https://bioinformatics.psb.ugent.be/webtools/Venn/). The number of targets predicted by the intersection of Target Scan (TS) miRmap (MM) and miRWalk was 12; the intersection of TS and MM revealed 150 targets, the intersection of MM and MW revealed 42 targets, while the intersection of MW and TS revealed 28 targets.

### KEGG pathway enrichment and gene ontology analysis for miR-195-5p target genes

KEGG pathway enrichment for miR-195-5p target genes indicated that they are predominantly involved in the PI3K-Akt signaling pathway (*FGF7, AKT3, FGF2* and *VEGFA*.; *P* = 0.031), Ubiquitin mediated proteolysis (*CDC23, CUL2* and *CDC27*; *P* = 0.029), MAPK signaling pathway (*FGF7, MAPK8, MKNK1* and *AKT3*; *P* = 0.019), Cell cycle (*CDC23, YWHAQ, CHEK1* and *CDC27*; *P*  = 0.015) and mTOR signaling pathway (*AKT3* and *SGK1*; *P* = 0.002) (Fig. 3B, Supplementary Table S4). The GO analysis for miR-195-5p revealed that its target genes were enriched in biological processes (BPs) such as ubiquitin-dependent protein catabolic process (*CUL2* and *UBE4B*; *P* = 0.006), cell division (*CDC23* and *CDC27*; *P* = 0.011), mitotic cell cycle (*WEE1* and *MYB*; *P* = 0.013) (Fig. 3C, Supplementary Table S5). The molecular functions (MFs) of these genes were protein serine/threonine kinase activity (*MAPK8, MKNK1, AKT3, CHEK1,* and *SGK1*; p = 0.011), GTP binding (*GLUD1* and *RRAGA*; p = 0.018) and ubiquitin-protein ligase activity (*WWP1* and *BTRC*; p = 0.009), (Fig. 3E, Supplementary Table S5). The analysis of cellular component (CC) Gene Ontology terms revealed significant enrichments in specific cellular locations for the genes. Notably, FGF2, FGF7, and AKT3 were found to be significantly associated with cytosolic localization (*P* = 3.9e−04), whereas CHEK1, CDC27, and MKNK1 exhibited significant enrichment in the nucleus (*P* = 1.4e−03) (Fig. 3D, Supplementary Table S5).

### Isolation and molecular characterization of endometrial primary cells

**Figure 4:**
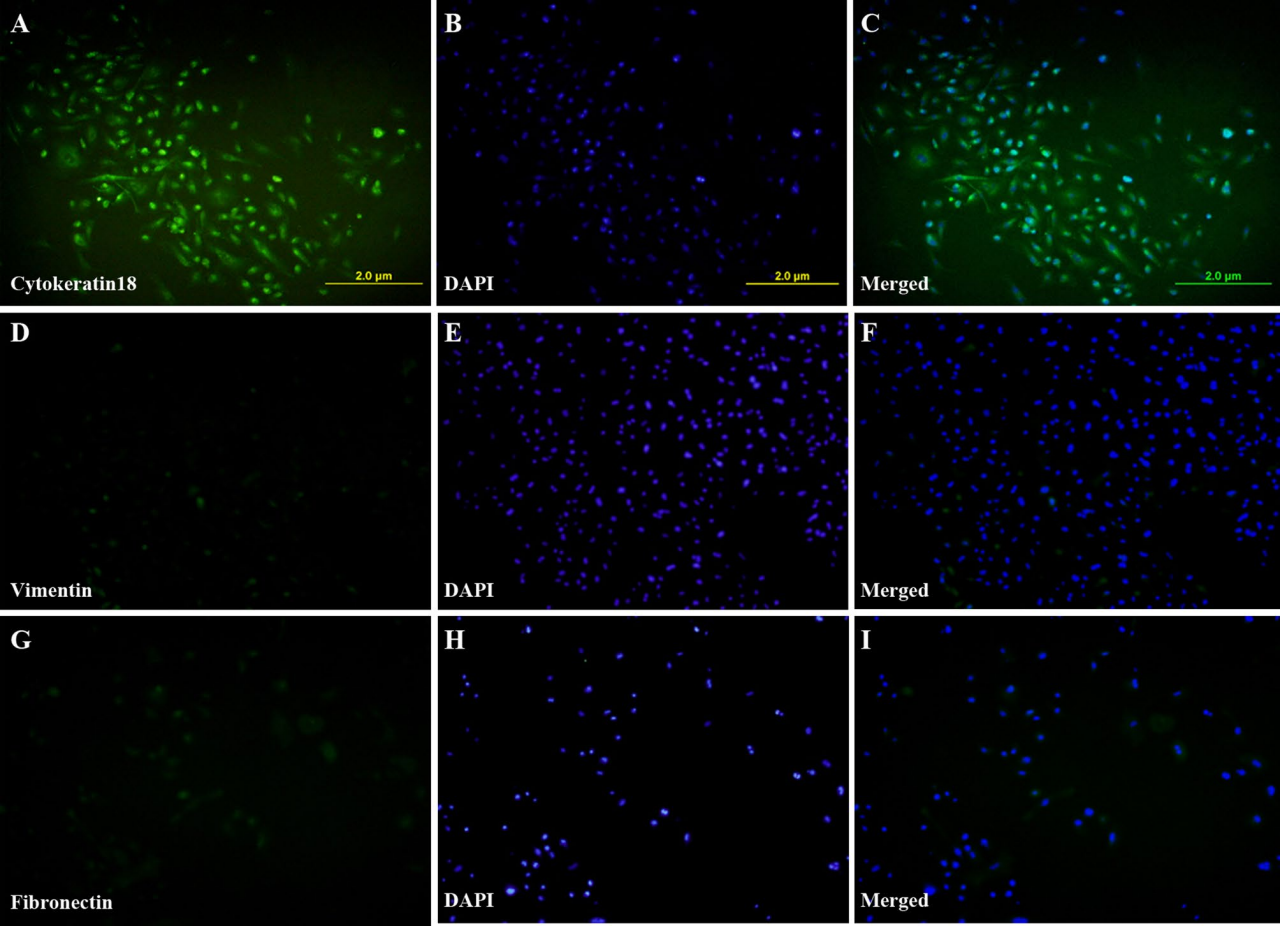
Primary endometrial cell identification. Isolated epithelial cells were stained with (**A**–**C**) epithelial cell marker cytokeratin18 and (**D**–**F**) stromal cell marker vimentin and (**G**–**I**) epithelial to mesenchymal transition marker Fibronectin to demonstrate that the majority of the cells are epithelial endometrial cells.

The cells isolated from the uterine horns contained most of endometrial epithelial cells (EECs) with some impurities of stromal cells. Nonetheless, seeding the isolated cell population for 12 h in a culture flask effectively separated the pure epithelial cells from the stromal cells. After isolation, EECs could be differentiated from stromal cells by their large and spherical appearance. The EECs remained in the suspension while the stromal cells got attached to the substratum (culture flask) after 12 h. Endometrial epithelial cells showed cuboidal or columnar morphologies^27^ and gradually became a squamous polygon, whereas stromal cells possessed a spindle shape. EECs had granular cytoplasm and big, centrally located nuclei (Supplementary Fig. S2). In addition, immunocytochemistry (ICC) and PCR determined the purity of epithelial cells. Vimentin, cytokeratin18, and fibronectin are the characteristic markers for endometrial stromal cells, endometrial epithelial cells, and epithelium to mesenchymal transition cells, respectively, and these were used to screen the cultured cells. A strong signal for cytokeratin 18 and a faint signal for vimentin and fibronectin indicated that the cell population consisted predominantly of EECs (Fig. 4).

### Expression profile of miR-195-5p target genes upon endometrial primary cells transfection with miR-195-5p mimic

**Figure 5:**
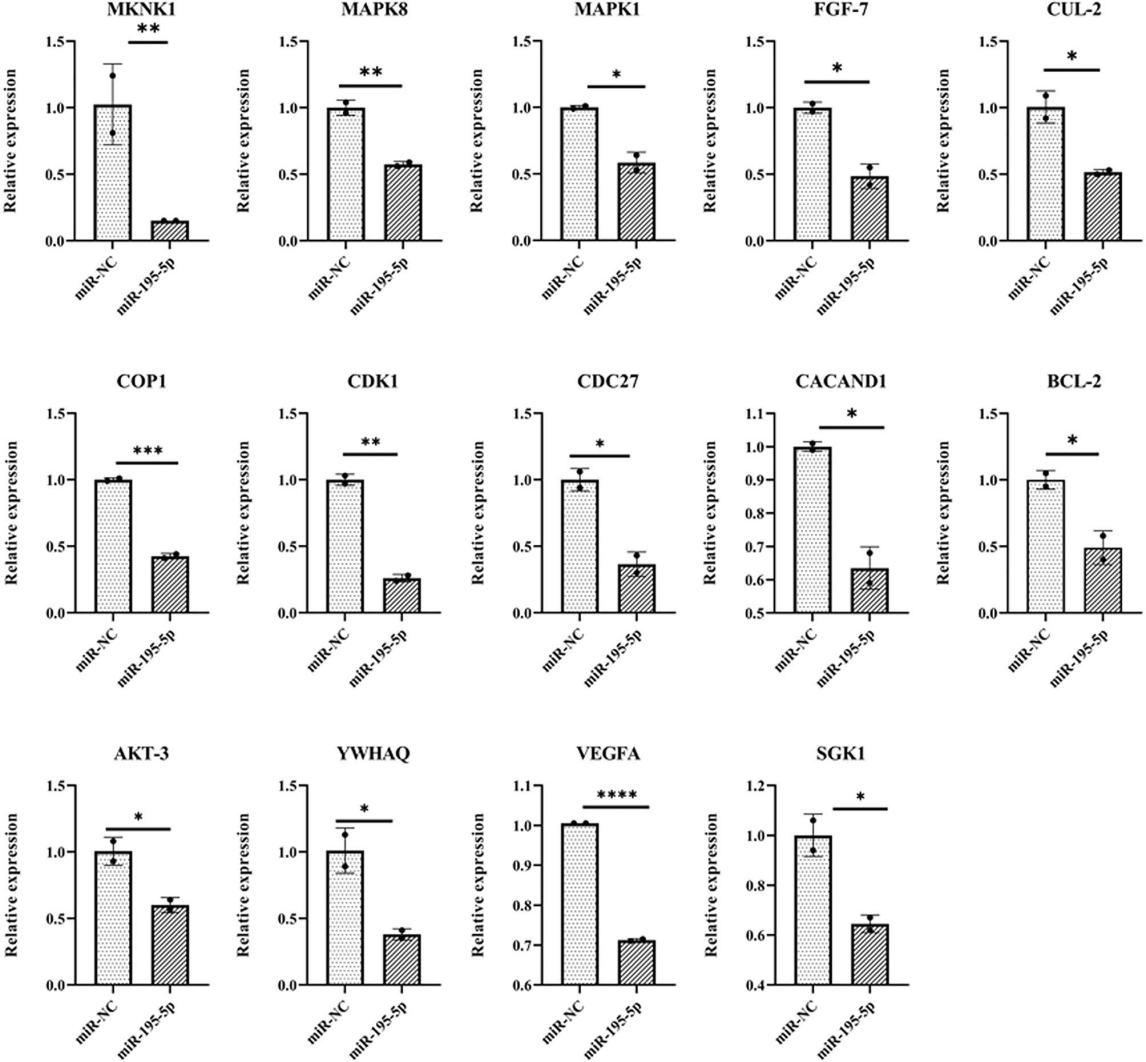
Relative expression patterns of miR-195-5p target genes. The relative expression profiles of the miR-195-5p target genes in the buffalo endometrial primary cells transfected with miR-195-5p mimic and miR-NC. Expression values were normalized to GAPDH (n = 2). Transfected cells are identified on the X-axis. Y –axis represents relative expression levels. *P < 0.05; **P < 0.01; ***P < 0.001 and ****P < 0.0001.

To further investigate the role of miR-195-5p in early pregnancy establishment, the endometrial primary cells were transfected with the miR-195-5p mimic. The relative expression of the target genes between the cells transfected with miR-195-5p mimic and miR-NC (miRNA negative control) was determined upon transfection (n = 2). The relative expression profile was generated using qRT-PCR for the miR-195-5p target genes involved in the (i) cell cycle (*YWHAQ, CDC27, CHEK1, CDC23,* and *SMAD2*) (ii) PI3-Akt signaling (*AKT-3, FGF-7, YWHAQ, MAPK8, SGK1, VEGFA, MKNK1, MAPK8, CACAND1,* and *MYB*), (iii) Ubiquitin mediated proteolysis (*CUL-2, CDC27, WWP1, BTRC, CDC23,* and *UBE4B*) (iv) MAPK pathway (*AKT-3, MKNK1, CACNA2D1, FGF-7,* and *MAPK8*) and (v) mTOR signaling pathway (*AKT-3* and *SGK1*). The relative expression of the miR-195-5p target genes such as *YWHAQ, CDC27, AKT-3, FGF-7, MAPK8, SGK1, VEGFA, CACAND1, CUL2, MKNK1,* and *CACAN2D1* were found to be significantly (*P* < *0.05*) suppressed. At the same time, no significant differences were observed in *SMAD2, CHEK1, CDC23, MYB, WWP1, BTRC,* and *UBE4B*. In addition to miR-195-5p target genes, the relative expression of *CDK1, CDKN1A, CDKN1B, COP1, CREB1, BCL-2, CCNG2, RAF1,* and *MAPK1* genes involved in the pathways as mentioned above were also measured. The relative expression of *CDK1, COP1, BCL-2,* and *MAPK1* genes was found to be significantly decreased, while no significant changes were observed in the expression of *CDKN1A, CDKN1B, CREB1, RAF1, GRB2,* and *CCNG2*. The fold changes in the expression levels of the genes, as mentioned above, between the miR-NC and miR-195-5p transfected groups are given in Fig. 5, Supplementary Fig. S4, and Table 1.

**Table 1:** Relative expression of the studied genes in terms of fold change.

S. No	Gene name	Fold change	Pathway
1	CDK1	− 0.77	Cell cycle, PI3-Akt signaling
2	YWHAQ	− 0.63	Cell cycle, PI3-Akt signaling
3	CDC27	− 0.64	Cell cycle, Ubiquitin mediated proteolysis,
4	AKT-3	− 0.41	PI3-Akt signaling, MAPK pathway, mTOR signaling pathway
5	FGF-7	− 0.52	PI3-Akt signaling, MAPK pathway
6	COP-1	− 0.58	PI3-Akt signaling, Ubiquitin mediated proteolysis
7	MAPK8	− 0.43	PI3-Akt signaling, MAPK pathway
8	SGK1	− 0.36	PI3-Akt signaling, mTOR signaling pathway
10	BCL-2	− 0.51	PI3-Akt signaling
11	MAPK1	− 0.42	PI3-Akt signaling
12	VEGFA	− 0.29	PI3-Akt signaling
13	CACNA2D1	− 0.37	MAPK pathway
14	CUL2	− 0.49	Ubiquitin mediated proteolysis,
15	MKNK1	− 0.88	PI3-Akt signaling, MAPK pathway,
17	CHEK1	− 0.40	Cell cycle,
18	CDKN1A	− 0.46	Cell cycle, PI3-Akt signaling
19	CDC23	− 0.48	Cell cycle, Ubiquitin mediated proteolysis
20	CDKN1B	− 0.34	Cell cycle
22	MYB	− 0.02	PI3-Akt signaling
24	CREB1	− 0.17	PI3-Akt signaling
25	RAF1	− 0.11	PI3-Akt signaling
26	CCNG2	0.12	PI3-Akt signaling
27	WWP1	− 0.15	Ubiquitin mediated proteolysis
28	BTRC	− 0.65	Ubiquitin mediated proteolysis
29	UBE4B	− 0.23	Ubiquitin mediated proteolysis
30	GRB2	− 0.245	MAPK, PI3-Akt signaling
31	SMAD2	0.5	Cell cycle

### Quantification of cell proliferation and apoptosis after transfection of the endometrial primary cells with miR-195-5p mimic

**Figure 6:**
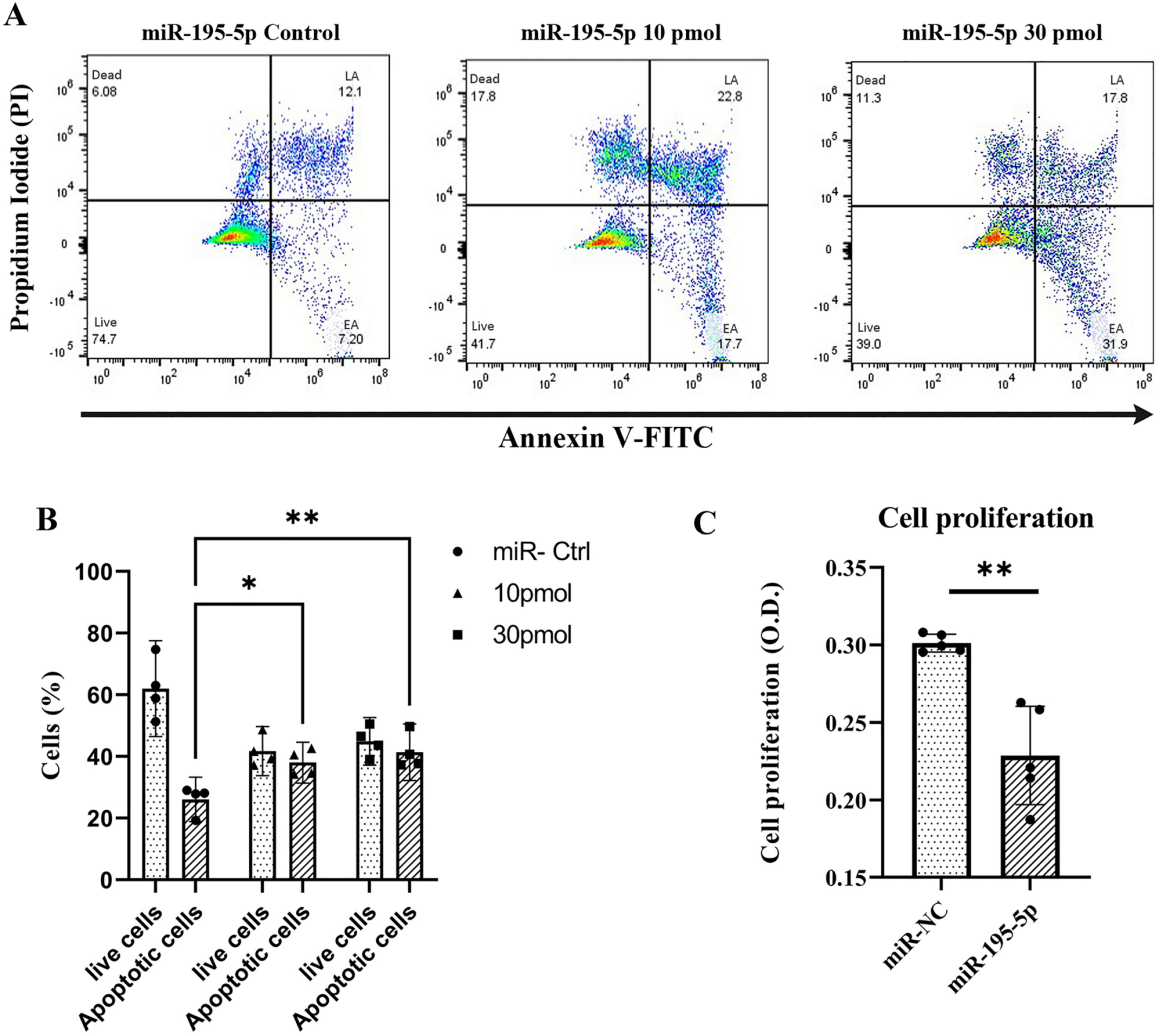
(**A**) Cellular apoptosis assay with FCM. (**A**) Flow cytometry analysis with Annexin V-FITC/Propidium Iodide (PI) staining was performed to evaluate the percentage of apoptotic cells in endometrial primary cells transfected with miR-negative control and miR-195-5p mimic at a concentration of 10 pmol, 30 pmol (**B**) The percentage of apoptotic cells in the miR-195-5p transfected group were significantly increased compared with that of miRNA negative control (n = 4). (**C**) Cell proliferation assay. BrdU assay was used to detect the proliferation level of cells in the miR-NC and miR-195 transfected group (n = 5). *P < 0.05; **P < 0.01.

Cell proliferation rate was measured post 48 h of miRNA transfection by BrdU incorporation to correlate the RT-qPCR results with cell proliferation and death. The cell proliferation rate was observed by calculating the mean of their optical densities. The mean optical density of the cells transfected with miR-195-5p was found to be significantly decreased (*P* < 0.05) compared to miR-NC transfected cells, or in other words, the BrdU incorporation into the proliferating cells was less in the miR-195-5p mimic transfected cells (n = 5) (Fig. 6C). To establish a correlation between the role of miR-195-5p and the regulation of apoptosis, the apoptotic rates were determined for the cells transfected with the miR-195-5p and miR-NC using flow cytometry-based Annexin V dead cell apoptosis detection kit. The data analysis revealed that the percentage of apoptotic was significantly higher *(P* < *0.01)* in the miR-195-5p transfected cells compared to the cells transfected with miRNA negative control transfected cells (n = 4) (Fig. 6A,B).

## Discussion

The aim of this study was to find the function of pregnancy-associated extracellular vesicle (EV) miRNAs in the maternal systemic circulation and assess their impact on shaping the uterine environment. In early pregnancy, there is a dynamic exchange of secretions between the developing conceptus and the endometrium, leading to intricate interactions that drive structural and functional changes in the endometrium. These changes enhance uterine receptivity for successful embryo implantation^28,29^.

Previous research has shown that circulatory miRNAs play a role in regulating pregnancy in cattle and buffalo. There is also emerging evidence suggesting the significance of EV-miRNAs in controlling embryo development, endometrial functions, and the communication between embryos and endometrium during pregnancy establishment^30–34^. In this study, we identified placental-origin EV-miRNAs in the blood of pregnant buffalo and investigated the functional impact of miR-195-5p on endometrial cells in vitro. Previous studies have reported differential expression of circulating extracellular miRNAs, including miR-195-5p, miR-200a-3p, miR-27, and miR-1246, in the blood plasma of pregnant cattle and buffaloes^31,35, 36^. Therefore, we examined the presence of these miRNAs within EVs derived from blood plasma and found that all four miRNAs were indeed present within these vesicles.

The reason for the presence of these miRNAs on the EVs may be attributed to the fact that EVs prevent the RNase-mediated degradation of the miRNAs^15,16^. We further investigated the differential expression of these EV-miRNAs among the pregnant and non-pregnant buffaloes concerning specific days post-artificial insemination. The expression of miR-195-5p was observed to be significantly high on day 19. Thus, we selected this miRNA for further experiments. In bovine species, day 19–20 of pregnancy is referred to as the implantation window, and the trophoblast attachment also begins in this phase^37^. It has been reported that a decrease in the miR-195-5p levels causes defective differentiation and invasion of the trophoblastic cells thus, leading to abnormal placentation in humans^38,39^. Therefore, miR-195-5p may have a crucial role in the implantation window of pregnancy. This miRNA may also diagnose pregnancy on day 19 due to its high expression.

The uterus is a dynamic physiological system in which cellular proliferation, differentiation, and apoptosis occur in a temporal and cell-specific manner during pregnancy. Decidualization of the endometrium cells is a vital event for successful embryo implantation. Studies have shown that regulating the cell cycle is extremely important for the decidualization of the endometrium^40^. The endometrial cells cease proliferating and become differentiated in the pre-implantation period. At the implantation site, the Endometrial cells undergo apoptosis when they come in contact with trophoblast^41,42^. Apoptosis is physiologically vital for optimal placental growth and development^43–45^. Due to high levels of miR-195-5p at day 19 post AI in pregnant buffaloes, we wanted to investigate its involvement in regulating cell proliferation in the endometrial cells.

KEGG pathway analysis identified that miR-195-5p's target genes are linked to key cell proliferation pathways vital for pregnancy establishment. The significant pathways include PI3K-Akt, Cell cycle, Ubiquitin-mediated proteolysis, MAPK, and mTOR signaling pathways. Gene Ontology analysis highlighted their involvement in Mitotic cell cycle, cell division, endocytosis, cell differentiation, and protein serine/threonine kinase activity. Specifically, the PI3-Akt pathway supports endometrial cell migration and decidualization during the implantation window^46,47^. Ubiquitin-related proteins play crucial roles in endometrial remodeling, placental development, and embryo implantation^48,49^. Inhibiting the mTOR pathway can induce cell death during implantation^50^. The MAP Kinase pathway regulates vital cellular functions such as proliferation, differentiation, apoptosis, and development^51^. Notably, the role of EV-miR-195-5p in modulating endometrial cells remains unexplored in existing literature.

The RT-qPCR results indicate a significant decrease in the expression of specific genes involved in various pathways following transfection with the miR-195-5p mimic in endometrial primary cells. These pathways include PI3-Akt signaling, Ubiquitin-mediated processes, MAPK signaling, Cell Cycle regulation, and mTOR signaling. Notably, suppression of the anti-apoptotic BCL-2 gene promotes apoptosis^52^. AKT, particularly AKT-3, is directly influenced by PI3K and impacts cell proliferation, differentiation, and apoptosis^53,54^. FGF-7 contributes to cell proliferation, differentiation, migration, and angiogenesis^55^. MAPKs regulate apoptosis through transcriptional and post-transcriptional mechanisms, sometimes acting as pro-apoptotic or anti-apoptotic in specific cell types^56^. YWHAQ protects cells from apoptosis by inhibiting p53 and Bax in hepatocellular carcinoma^57^. CDK1 inhibition induces apoptosis in primary effusion lymphoma cells^58^. Genes like SGK1, COP1, and CDC27 promote cell proliferation and regulate apoptosis in cancer cells^59–62^. VEGFA promotes vascular permeability and angiogenesis, affecting proliferation, migration, cell survival, and apoptosis inhibition in endothelial cells^63^. Cyclin D1 and CDKL1, both proto-oncogenes, regulate the G1 to S phase transition in the cell cycle, facilitating progression^64,65^. In summary, the RT-qPCR results show that miR-195-5p mimic transfection significantly alters gene expression, particularly in apoptosis and cell cycle regulation pathways, revealing its multifaceted impact on endometrial primary cells.

To investigate the link between miR-195-5p suppression and cell cycle regulation in endometrial primary cells, we conducted BrdU ELISA-based proliferation assays and flow cytometry-based apoptosis detection on cultured cells transfected with the miR-195-5p mimic. The transfection led to reduced cell proliferation (BrdU assay) and increased apoptosis (flow cytometry), consistent with previous findings indicating that elevated miR-195 levels decrease cell viability and proliferation while promoting apoptosis^66,67^. This suggests miR-195-5p's involvement in modulating apoptotic and proliferation pathways that could impact embryo implantation. Endometrial tissue remodeling during implantation involves apoptotic cell death, rendering the uterus receptive to the embryo^19,20^. Thus, our study sheds light on miR-195-5p's role in regulating these processes, potentially influencing embryo implantation. To the best of our knowledge, this is the first study highlighting the role of EV-borne miR-195-5p in governing apoptosis and cell proliferation in endometrial cells.

## Conclusion

**Figure 7:**
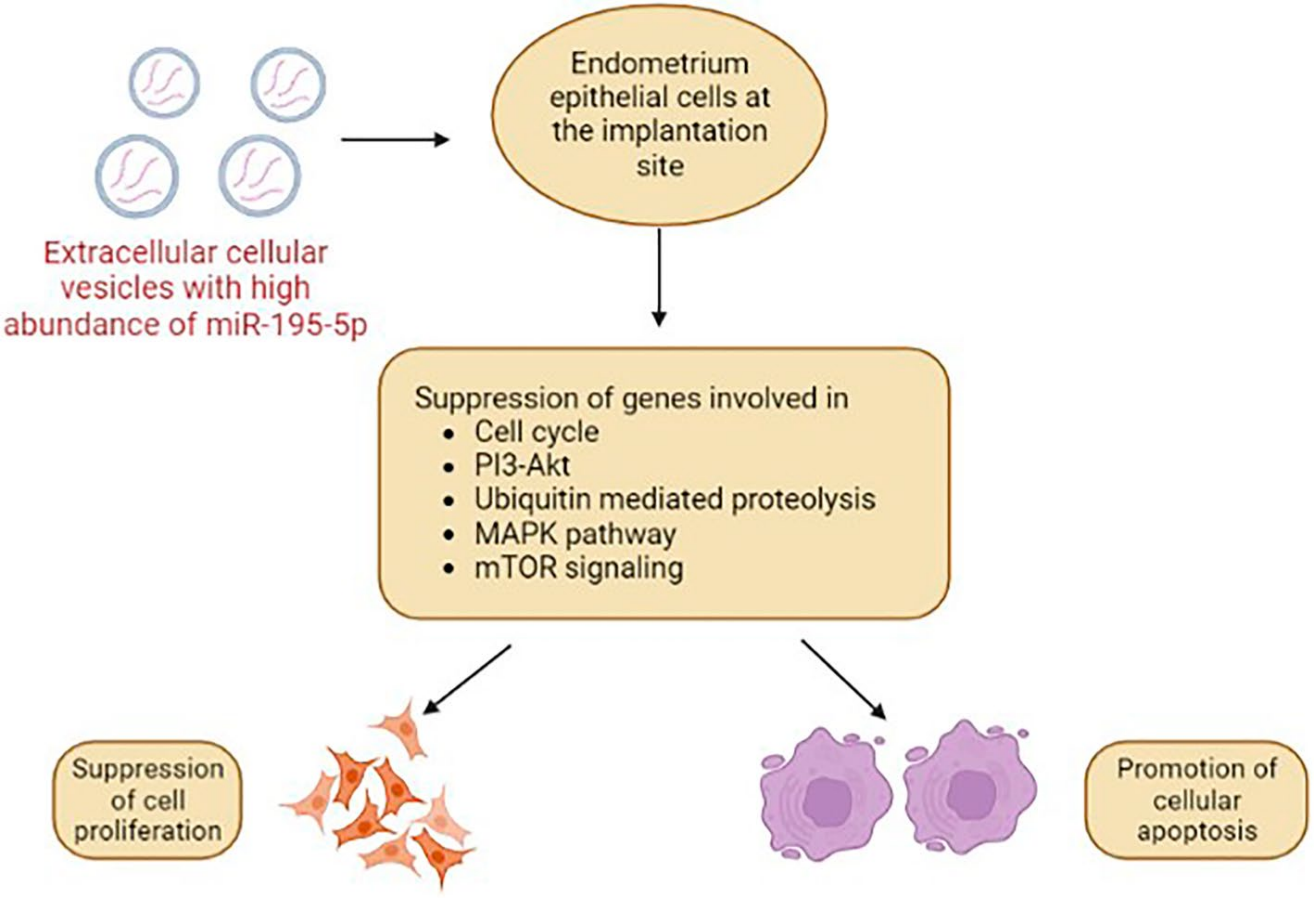
Schematic diagram representation of the effect of Extracellular vesicle-associated miR-195-5p on the endometrium primary cell culture.

In conclusion, the increased expression of miR-195-5p promotes apoptosis by regulating the expression of genes involved in cell proliferation and apoptosis at the initial stages of implantation (Fig. 7). This may have a profound role in promoting uterine receptivity; thus, modulation of miR-195-5p may enhance the chances of successful pregnancy in buffaloes. In addition, due to the high levels of miR-195-5p in the blood plasma of pregnant buffaloes at day 19 post-AI, it can be used as a candidate miRNA for detecting a successful pregnancy.

## Methods

### Ethics statement

The animals employed in the study were reviewed and approved by the Institutional Animal Ethics Committee, ICAR-National Dairy Research Institute, Karnal, India. The study was conducted in compliance with ARRIVE guidelines and the relevant guidelines and regulations performed on all methods^68^.

### Experimental design

This study was conducted through several experiments.

In experiment 1, Blood samples were collected from different animals categorized into two groups viz., pregnant and non-pregnant Murrah Buffaloes. For the characterization, EVs were isolated from the blood plasma of three different randomly selected Murrah buffaloes irrespective of their pregnancy status. EVs were characterized by the Differential light scattering method, Nano Sight, to determine the average concentration and size, western blotting (WB) to identify the presence of protein markers and visualized under transmission electron microscopy for morphological identification.

In Experiment 2, miRNAs were chosen using previous Next-Generation Sequencing (NGS) results, which highlighted their elevated presence during the initial stages of pregnancy in buffaloes (Accession: PRJNA705293, ID: 705293) as documented in NCBI. Additionally, miR-1246 was selected based on existing scholarly references. These specific miRNAs were quantified using RT-qPCR in the miRNA isolated from the blood plasma-derived extracellular vesicles (pregnant buffaloes = 3, non-pregnant buffaloes = 3). miRNA targets were identified, and pathway enrichment and gene ontology analysis were performed on the selected miRNA target genes.

In Experiment 3, The primary endometrium primary cells were cultured and transfected with miRNA mimic and negative control. The relative expression of the miR-195-5p target genes was analyzed through RT-qPCR, and the effect of miRNA transfection on cell proliferation and apoptosis was measured.

### Experimental animals and blood collection

Buffaloes falling under different experimental groups, viz., pregnant, cyclic non-pregnant animals (n = 3 each), were included for blood sample collection. In the first group (pregnant), blood samples were collected on Day 15 (counted from the day of insemination), Day 19, Day 25, and Day 30. Confirmation of pregnancy was done using trans-rectal ultrasonography on Day 100 of insemination. In the second group (non-pregnant), to preserve consistency with the blood collection timings in pregnant animals, cyclic buffaloes in this group were not inseminated at estrus. Blood samples were taken from these animals on Days 15, 19, 25, and 30 post-estrus. These animals displayed estrus 20–22 days following the previous estrus, however, this was not considered, and blood was collected up until Day 30 of the preceding estrus. In this scenario, Days 25 and 30 could correspond to Days 5 + /− 3 and Day 10 ± 3 of the second estrous cycle, respectively. However, because they were not bred during both estruses, the animals were not pregnant. As a result, they also reflect the samples that are not pregnant. Blood samples were collected from the jugular vein in the EDTA vacutainer (Becton Dickinson, USA) using 18 gauze syringes. All the procedures were performed at Livestock Research Center, NDRI, Karnal. Plasma was immediately isolated from the blood samples by centrifugation at 3000 g for 5 min at 4 °C and was stored at − 80 °C.

### Extracellular vesicle isolation

Stored plasma samples (80 μL) were thawed on ice and centrifuged at 10,000 g for 30 min to pellet down the cells, debris, and platelets. The supernatant was then passed through a 0.22 µm syringe filter (Millex-GV Syringe filter unit Millipore), and EVs were isolated from plasma using a miRCury exosome isolation kit (76603, Qiagen Hilden, Germany). In brief, the 600µL of the filtered plasma was treated with 6 µl of thrombin (500U/ml) (supplied with kit) and allowed to incubate for 5 min for de-fibrination followed by centrifugation at 10,000×*g*. The supernatant was collected, and 200 µl of precipitation buffer was added along with RNAse A (R6513, Sigma-Aldrich) for removal of miRNAs carried by lipoprotein, Argonaute protein, and other free miRNAs outside of EVs) at a concentration of 10 µg/ml, and allowed overnight incubation at 4 °C. The next day, RNAse inhibitor (N8080119, Applied Biosystems) was added to the reaction mixture at 150U/ml before precipitation of EVs by centrifugation at 500×*g* for 5 min at 20 °C. The EV pellet was resuspended into the 270 µl of the resuspension buffer.

### Characterization of EVs

EVs were isolated from blood plasma sample of three different randomly chosen animals irrespective of their pregnancy status as previously described and were pooled together. The isolated EVs were characterized through several approaches following MISEV 2018 guidelines:

#### Differential light scattering

Using a Zetasizer Nano ZS ZEN3600 (Malvern Instruments, Malvern, UK, the size distribution of EVs was determined. The scattered light's intensity was measured at 173°. The particle size measurement was made in triplicates at 25 °C followed by data analysis and processing which were carried out with the help of the Zetasizer software, version 7.03. After EV isolation, 10 μL of purified EVs were diluted in 990 μL of filtered 1X PBS and sonicated in an ultrasonic water bath for 1 min at 50 Hz. The sample was immediately put in a disposable cuvette for size measurements to avoid aggregation of the EVs. Three independent measurements were recorded for each sample.

#### Nanoparticle tracking assay

All particle tracking analyses used a Malvern NS300 device with a 488 nm laser and a 500 nm long-pass filter for fluorescence detection. To perform the NTA count, all samples were diluted in a ratio of 1:100 with 1X PBS (10010023, Gibco). The sample infusion pump was set to a constant flow rate of 5 μL/minute. The camera level was set at 14, as all particles were visible at this level without signal saturation, and the detection threshold was set at 5. The ideal measurement concentration was found by pre-testing the ideal particles per frame value (40–120 particles/frame). The sample was measured five times, and 30–60 s videos were collected with a minimum of 200 valid tracks recorded per video. Then the particles tracked were calculated and graphed using GraphPad Prism. All studies were performed on Nanosight 3.0 software using the default settings.

#### Transmission electron microscopy

Isolated EVs were diluted 1:100 in 1X PBS and fixed with an equal volume of 2% paraformaldehyde prepared in phosphate buffer for 1 h at 4 °C. The carbon Formvar film-coated 300-mesh transmission electron microscopy grids were glow-discharged. The fixed EV samples were applied to the TEM grid and incubated for 15 min at room temperature. After washing with PBS, the samples were fixed with 1% glutaraldehyde for 5 min. Upon washing with distilled water, the grids were stained with 1% phosphotungstic acid for 45 s, wicked off with Whatman filter paper, and allowed to dry before viewing. TEM examination was performed using JEM1400 FLASH transmission electron microscope (JEOL USA Inc, Peabody, MA, United States) at 120 kV and viewed under 250,000× magnification with highly sensitive olympus sCMOS camera at the TEM facility of Advanced Technology Platform Centre (ATPC), Regional Centre for Biotechnology, Faridabad.

#### Western blotting

Protein was isolated by incubating the isolated EVs in RIPA buffer (Sigma-Aldrich) for 15 min at room temperature, followed by sonication. Approximately 20 µg of protein was loaded per well into SDS-PAGE gel, and proteins were separated by SDS polyacrylamide gel electrophoresis using a mini gel tank electrophoresis system (Invitrogen Life Technologies) and then transferred to Immobilon-FL polyvinylidene difluoride membranes (Millipore, Billerica, MA, USA). The membrane containing the transferred protein was probed with primary mouse polyclonal anti-TSG-101 (1:1000 SC-7964, Santa Cruz Biotechnology, USA) primary goat anti-CD63 (1:3000, STJ140029, St. Johns Laboratory, London, United Kingdom), primary CD9 Monoclonal Antibody (IVA50) (1:6000, MA1-19301, Invitrogen) and primary Anti-Calnexin (CNX) Monoclonal Antibody (CAA280Hu22, Cloud Clone Corp, USA)^69^. Membranes were washed in Tris-buffered saline (TBS- pH 7.6) and incubated for 2 h in TBST (TBS with Tween-20) containing 5% BSA along with horseradish peroxidase-conjugated anti-mouse secondary antibody (1:10000, SC-516102, Santa Cruz Biotechnology), anti-goat secondary antibody (1:20000, STJ99512, St. Johns laboratory), anti-mouse secondary antibody ( for CD9-1:30000, for Calnexin- 1:10000, A9044, Sigma) respectively. The Substrate Pierce ECL Western Blotting kit (32106) was utilized in the next step for chemiluminescence, and subsequently, the membranes were exposed to X-ray film for 1–5 min before visualization.

### Isolation of miRNA and cDNA synthesis

Small EVs were isolated from the pregnant animals (n = 3) at days 15, 19, 25 and 30 post-AI and non-pregnant animals (n = 3) as previously described. The miRNA was isolated from the EVs using Qiagen Advanced miRNeasy Serum/plasma kit (cat. 217204, Qiagen, Germany) according to the manufacturer’s instructions with the addition of bacteriophage MS2 RNA (10165948001, Roche) as a carrier RNA (to improve the RNA yield) in 20 μL volume. *C.elegans* miRNA, cel-miR-39-3p, was used as an exogenous spike-in control. The RNA concentration for each sample was measured using NanoDrop 1000 spectrophotometer (NanoDrop Technologies), and a 10–15 ng/µl concentration range was obtained from each sample. Approximately 120 ng of the isolated RNA was converted into cDNA for each sample using the Reverse Transcriptase Kit II miScript (Cat# 218161 Qiagen, Valencia, CA) using HiSpec buffer (5X), miScript nucleic acid mix, and miScript enzyme (RT) in a final reaction volume of 20 µL for mature miRNA profiling. The samples were incubated at 37 °C for 1 h and then at 95 °C for 5 min to inactivate the miScript enzyme (RT). Subsequently, the cDNA was diluted in nuclease-free water (2.5 ng/µl for qPCR) and stored at − 20 °C.

### Primer designing for miRNA

Specific miRNAs were selected based on the prior NGS data that indicated their highest abundance at the stage of early pregnancy in buffaloes^31^, NCBI, Accession: PRJNA705293, ID: 705293); while miR-1246 was selected based on prior literature^36^. A total of four miRNAs, i.e., miR-27, miR-1246, miR-195-5p, and miR-200a-3p were selected, and miR-26a, U6, let-7a, miR-23a, and miR-16a were initially chosen as reference controls. Primers were designed using the miR primer tool^70^. The forward primers were made against the seed sequence of the miRNAs from the database available at http://www.mirbase.org/. The reference controls miR-23a, miR-26a, and U6 were later excluded due to their lesser stability in different biological samples as calculated by the online tool RefFinder (http://www.heartcure.com.au/reffinder/). The mature miRNAs and the primer sequences utilized for the q-PCR assay have been listed in Supplementary Table S1. Primer oligos were procured from Sigma-Aldrich.

### miRNA quantification using quantitative Real Time-PCR (RT-qPCR)

Relative quantification of selected miRNAs was performed using miScript SYBR Green PCR Kit (QIAGEN, Valencia, CA, USA). Approximately 5 ng cDNA was used as a template in each reaction, and *C*. *elegans* miRNA cel-miR-39-3p was used as an exogenous control. A no-template control (NTC) was also run on each plate. The non-pregnant group's sample from day 15 post-AI was taken as the calibrator. The reactions were performed in duplicates using the Bio-Rad c1000 thermal cycler Detection System (USA) with the following conditions: 95 °C for 5 min, 45 amplification cycles consisting of incubations at 94 °C for 15 s, 55 °C for 45 s and 70 °C for 30 s, followed by melt curve analysis from 65 to 95 °C. The relative expression of the target miRNAs was calculated using the 2^−ΔΔCT^ method^71^ by considering let-7a and miR-16a as reference controls. The differential expression levels of miRNAs among the non-pregnant and the pregnant groups were analyzed by two-way ANOVA and Dunnett’s multiple comparison test using the GraphPad Prism 9.0 (GraphPad Software, La Jolla California USA) software.

### miRNA target prediction, KEGG pathway enrichment, and Gene Ontology analysis

For miRNA target prediction, TargetScan (http://www.targetscan.org/), miRmap (https://mirmap.ezlab.org/), and miRwalk (http://mirwalk.umm.uni-heidelberg.de/) tools were used which are freely available online. Target scan and miRmap consider the seed-based interaction in the 3′UTR of the target mRNA. Only the top predicted targets from each database were selected to maximize the accuracy of results. We selected miR-195-5p for target prediction by combining the results of different target prediction tools to look for common predicted targets among the tools^72,73^. We considered only those targets predicted by at least two of the three tools and proceeded with them for further analysis. Gene ontology and KEGG pathway enrichment analysis were performed for the selected miRNA target genes using the Database for Annotation, Visualization, and Integrated Discovery (DAVID)^67^. Pathways and GO term comprising the highest number of genes with a p-value threshold of 0.05 were considered significant^74–76^.

### Isolation of endometrial primary cells

The procedure for the isolation of endometrial primary cells has been described in supplementary information.

### Characterization of endometrial primary cells by immunocytochemistry

The cells were seeded in a 12-well cell culture plate (Nunc, Thermo Scientific) with a seeding density of 0.1 × 10^6^ cells and cultured up to 70–80% confluency. The culture medium was removed, and the cells were washed twice in 1X PBS and fixed in 4% paraformaldehyde for 20 min at room temperature. The cells were washed twice with 1X PBS and then incubated with antigen retrieval buffer (100 mM Tris, 5% w/v Urea, pH-9.5) for 10 min at 95 °C, followed by washing with 1X PBS. The cells were permeabilized by incubating in a permeabilization buffer (0.1% Triton X-100) for 15 min at RT. The cells were then washed with 1X PBS thrice, and the plate surface was blocked with blocking buffer (5% BSA in PBS) for 1 h at room temperature. The cells were later incubated with primary anti-mouse antibodies (1:200 dilution) against Cytokeratin18 (SC-32329, Santa Cruz Biotechnology), Vimentin (Invitrogen MA5-11883), and Fibronectin (SAB4200845, Sigma Aldrich). The cells were then washed thrice with PBST and incubated with FITC conjugated goat anti-mouse IgG secondary antibody (1:500 dilution; F9137, Sigma Aldrich) in the dark for 1 h at RT followed by three times washing with 1X PBST. The nuclei were stained with DAPI (Sigma Aldrich) at a working concentration of 1 µg/mL for 10 min at RT. Finally, the cells were again washed with PBS, and one drop of prolong gold antifade (P36934, Thermofisher Scientific) was mounted. Cells were observed under a BX-51 Olympus fluorescence microscope at 200× magnification using excitation wavelengths for FITC (495 nm) and DAPI (358 nm).

### Transfection of miRNA mimics into endometrial primary cells

Cells were seeded at a density of 2–3 × 10^5^ cells per well into a 6-well cell culture plate. After the cells attained 70–80% confluency, the medium in each well was removed and replaced carefully with 200 µL of the serum-free opti-MEM medium (51985034, Gibco). The cells were then transfected with 20 pmol miRNA mimic miR-195-5p and miRNA negative control mimic (mir-Vana™). Transfection mixtures were prepared according to the manufacturer’s protocol.

The media was replaced with complete DMEM post 6 h of transfection. The cells were harvested in TRIzol reagent (Invitrogen) after 48 h of transfection and stored at − 80 °C until further use.

### RNA isolation from the cultured cells and cDNA synthesis

Total RNA was isolated from the cells harvested in TRIzol and quantified using NanoDrop ND-1000 UV–visible spectrophotometer. To evaluate the quality of the extracted RNA, 200 ng of the RNA was run in non-denaturing 1X TAE buffered 1% agarose gel (Sigma-Aldrich, USA). Two intact bands of ribosomal RNAs were looked for in the electrophoresed samples. The extracted RNA was treated with DNase I (Thermo Scientific, USA) to remove gDNA contamination by the manufacturer’s instructions. Approximately 500 ng of the isolated RNA were converted into cDNA using the RevertAid H minus first-strand cDNA synthesis kit (Thermo Scientific, USA).

### Primer design for miR-195-5p target genes

The primer design tool at NCBI, the Primer-BLAST was used to design primers for miR-195-5p target genes and four reference genes viz. *GAPDH, SUZ12, RPL19,* and *SLC30A6* from the CDS regions are available on the NCBI site. Primers spanning only the exon-exon regions were constructed wherever possible. Using the Oligo Nucleotide Properties Calculator, the primer’s self-annealing sites, mismatches, and secondary structures were analyzed (Kibbe, 2007). Before commercial synthesis, each set of primers was subjected to an additional check for specificity using the BLAST alignment tool (www.ncbi.nlm.nih/gov/BLAST) and in silico PCR (Kuhn et al. 2013). Sigma-Aldrich, USA commercially synthesized all the primers. The primer sequences used for q-PCR have been listed in Supplementary Table S2. *SUZ18, RPL19* and *SLC30A6* were later taken out of the study due to their sub-optimal efficiencies.

### RT-qPCR assay

Using the iTaq universal SYBR green supermix (Biorad, USA) in a 10 uL reaction, the relative quantification of the chosen miRNA target genes was carried out on a Biorad C1000 touch thermal cycler platform. Two technical replicates were employed for each reaction in the qPCR. The reaction conditions included initial denaturation of 95 °C for 10 min followed by 40 cycles of denaturation at 95 °C for 15 s, annealing at various optimized temperatures for 20 s, and extension at 72 °C for 20 s. The melt curve protocol included heating for 10 s at 95 °C and then 60 s for each 0.5 °C increment from 65 to 95 °C. Each plate was run with a no-template control (NTC) to ensure no nucleic acid contamination. The melt curve analysis was carried out to assure the formation of unique and specific products without primer dimers. The mean sample Cq (quantification cycle) values for miR-195-5p and miRNA negative control (miR-NC) transfected cells samples were calculated, and the differential gene expression was calculated using the 2^−ΔΔCT^ method as described previously using the *GAPDH* reference gene. The miR-NC transfected cells were taken as the calibrator. Statistical analysis was performed using the unpaired t-test as implemented in the GraphPad Prism 9.0.9 and a *P* value < 0.05 was considered to be statistically significant.

### Cell proliferation assay

Cell proliferation analysis was performed using Bromodeoxyuridine (BrdU) incorporation assay (Roche, Indianapolis, IN, USA). Cells were cultured on a 96-well plate at a seeding density of 1 × 10^4^ cells/well using complete media. They were subsequently transfected with the miR-195-5p mimic along with miRNA negative control as described previously, followed by 48-h incubation. The BrdU staining was performed according to the manufacturer’s instructions. The plate was read on an Infinite® 200 PRO NanoQuant (Tecan Trading AG, Switzerland); within 30 min of adding the substrate solution to the cells. The wells containing BrdU without the cells served as background control. The cell proliferation rate was observed by calculating their respective mean of the optical densities. The background values were removed from the initial values to acquire accurate BrdU uptake results. The experiment was performed in five replicates and the data was normalized to the miRNA negative control transfected cells. Statistical analysis was performed using the unpaired t-test as implemented in the GraphPad Prism 9.0.9 and a *P*-value < 0.05 was considered statistically significant.

### Annexin-V staining and flow cytometry

Cell apoptosis was assessed using a Dead Cell Apoptosis kit with Annexin V-Alexa Fluor 488 and propidium iodide dye (V13241, Invitrogen) according to the manufacturer’s protocol and the apoptotic rate was analyzed using flow cytometer (BD Flow Accuri C6) by considering 25,000 events. Data were analyzed using FlowJo software version 10.8.1 (Tree Star, Inc). Cells were cultured on a 12-well plate at a seeding density of 1 × 10^5^ cells/well. Following 48 h of miRNA mimics transfection as described previously, cells were harvested and washed 2–3 times with 1X PBS at 37 °C for 5 min. Subsequently, the cells were resuspended at 1 × 10^6^ cells/mL density, stained with 5μL Annexin V‑ Alexa Fluor 488 and 1μL (100 μg/mL) propidium iodide (PI) working solution in binding buffer (included in the kit) at room temperature for 15 min (n = 4). The apoptotic cells were measured using 499 nm excitation and 535 nm emission for Annexin V‑ Alexa fluor 488 detection while for propidium iodide, 521 nm excitation and 617 nm emission filter was used. Statistical analysis was performed using Šídák’s multiple comparisons test as implemented in the GraphPad Prism 9.0.9 and a *P* value < 0.05 was considered to be statistically significant.

### Supplementary Information

Supplementary Information 1.

Supplementary Table 5.

Supplementary Video 1.

## Data Availability

The datasets supporting the conclusions of this article are included within the article and its supporting information. Publicly available datasets were analyzed in this study. This data can be found here: https://www.ncbi.nlm.nih.gov/bioproject/705293PRJNA705293.
