# Supplementary material for: Circulatory extracellular vesicle derived miR-195-5p promotes cellular apoptosis and suppresses cell proliferation in the buffalo endometrial primary cell culture
Source: Sci Rep. 2023 Oct 4;13:16703. doi: 10.1038/s41598-023-43530-y (PMC10551009; doi:10.1038/s41598-023-43530-y)
Supplement: Supplementary file 1 — Supplementary Information 1. [file 41598_2023_43530_MOESM1_ESM.pdf]

Circulatory extracellular vesicle derived miR-195-5p promotes cellular apoptosis and suppresses cell proliferation in the buffalo endometrial primary cell culture.

Ankit Pal[1], Seema Karanwal[1], Jatinder Singh Chera[1] Vipul Batra[1], Arumugam Kumaresan[2], Parul Sarwalia [1], Tirtha K Datta[1], Rakesh Kumar[1*]

[1]Animal Genomics Laboratory, Animal Biotechnology Centre, National Dairy Research Institute, Karnal, India

[2]Theriogenelogy Laboratory, SRS of National Dairy Research Institute, Bengaluru, India

***Corresponding author: rakeshcift@gmail.com**

Supplementary Table S1: miRNA primer sequence

Chromosome	miRNA	Mature Sequence	Primer Sequence
Chrm 3	miR-27	UUCACAGUGGCUAAGUUCUGC	F: TTCACAGTGGCTAAGTTCTGC<br>R: miScript Universal Primer<br>(sequence is proprietary)
Chrm 3	miR-195-5p	UAGCAGCACAGAAAUAUUGGCA	F: TAGCAGCACAGAAATATTGGC<br>R: miScript Universal Primer<br>(sequence is proprietary)
Chrm 5	miR-200a-3p	UAACACUGUCUGGUAACGAUGU<br>U	F: TAACACTGTCTGGTAACGATGTT<br>R: miScript Universal Primer<br>(sequence is proprietary)
Chrm 1	bta-miR-1246	AAUGGAUUUUUGGAGCAGG	F: AATGGATTTTTGGAGCAGG<br>R: miScript Universal Primer<br>(sequence is proprietary)

Supplementary Table S2: Sequence of the primers for miRNA target genes and reference genes used for expression analysis

Primer	Sequence (5'⟶3')	Length	Tm	GC%
VEGFA	TCGAGACCCTGGTGGACATC	20	60.97	60
MAPK8	AAAGCCAGTCAGGCAAGGG	19	60.23	57.89
YWHAQ	CCTACAAGAACGTGGTCGGG	20	60.39	60
CREB1	AGGGCCTGCAGACATTAACC	20	60.03	55
FGF7	CATGACTCCAGAGCAAATGGC	21	59.6	52.38
MYB	AGCAGGCGCTTCCAACAC	18	60.98	61.11
CCNG2	GATCGTTTCAAGGCGCACAG	20	60.18	55
CDKN1A	CACTTGGACCTGTCGCTGTC	20	60.95	60
AKT3	CAGAACGACCAAAGCCAAACAC	22	60.79	50
MKNK1	CAGAGCTGACTACTCCATGCG	21	60.54	57.14
WWP1	ACAAACGGCAGCTCATCTCC	20	60.67	55
BTRCF	TCCTGGCTGCTTAAGTGCTC	20	60.04	55
CDC23F	CGCCGCCTATTACAGAGGAAG	21	60.6	57.14
CDC27F	GAAAGCAGCAGCAGAAGGTTTG	22	60.86	50
CUL2F	GTGTGGCCTATCCTGAACCC	20	60.11	60
COP1F/ RFWD2F	TCAGTGAGTAGCACCAATGGC	21	60.34	52.38
UBE4BF	CCCATAGCAGCATCAGCTCC	20	60.61	60
SMAD2F	GGGAGCAGAATACCGAAGGC	20	60.53	60
BCL2F	TGGAGGAGCTCTTCAGGGAC	20	60.62	60
RAF1F	TTCCACTGTTGGTGATGGCG	20	60.89	55
CDKN1BF	CGACCTGCCGCAGATGATTC	20	61.49	60
MAPK1F	GACCACACAGGGTTCCTGAC	20	60.25	60
CDK1F	TTCAGAGCTTTGGGCACTCC	20	60.25	55
CACNA2D1F	GTGGAGCCAAATAACGCACG	20	60.18	55
CHEK1F	TCGTTATGGGACACCAGCCC	20	61.91	60

SGK1F	AACCCAAATGTGAGCGGACC	20	60.89	55
GAPDHF	GCCTGGAGAAACCTGCCAAG	20	61.25	60
SUZ12F	TGGGGAATATGAAGTAGCCATGC	23	60.5	47.83
RPL19F	TCGAATGCCCGAGAAGGTAAC	21	60.13	52.38
SLC30A6F	TGGCTTCCTGCTAATGTGGTG	21	60.61	52.38

Supplementary Table S3: List of miRNA target predicted by various tools

Name of Target prediction tool	Targets count	Genes
Targetscan, miRmap miRwalk	12	MBNL2, MBNL2, SLC2A3, ACVR2A, TSPYL2, COPS2, ZNF362, PID1, PTH, FNTA, DPY19L4, NUDT4
Target scan miRmap	138	EIF2B5, GSKIP, VPS4A, MON1B, FNBP1L, SPTLC1, ARL2, GNAI3 PROSC, SERINC3, STRADB, RFWD2, USP6NL, PHF20, SCOC, DCP1A PDCD4, MKNK1, RAD23B, RNF217, SORT1, CHEK1, YWHAQ, ATP1B4 USP19, ECEL1, DLL1, RASEF, ASH1L, RBM6, ENTPD7, KIAA0226L, AKIRIN1, AP2A1, SMPD1, SYNRG, ARPP19, SGK1, TUBA1A, HOXA10 DOLPP1, EPHA70, PEX13, SCN4B, CACUL1, PPP2R5C, TFAP2D, WIPI2 TBC1D20, WBP11, SPRED1, SETD6, ATXN2, GABARAPL1, ZCCHC3, TMEM55B, WIF1, CSRNP1, CCNT2, SEH1L, TSPAN5, WEE1, FAM60A FERMT2, SLA2, CCDC19, CDC27, SYNDIG1, RPE, EZH1, KRT80, BACE1 PRSS12, AK4, FGF7. SESN1, RASGEF1B, SYT3, FLT3, ACSBG1, SLC9A6 TBPL1, MEOX2, ATXN7L3, BFAR, MAPK8, JARID2, KLHL18, C16orf72, PAFAH1B2, UBE4B, PPAP2B, RNF111, IST1, DYNC1I1, CCDC85C, SHOC2 SSR1, ARL3, SALL4, ZDHHC14, PPP6C, PDCD11, KBTBD2, BAG5, PDIA6 CDC23, AMOTL1, MIPOL1, GORASP2, RNF10, TMEM55A, RNF125, TTC14 SOCS5, UBE2V1, POLR3F, SH3GL2, RSPO3, TXN2, ZNF592, CNIH3, HTR2A, VTI1B, PLEKHA5, KRT85, HMGA1, KDSR, OMG, HSPG2, AKT3 LRIG2, ARMCX2, APP, RPS6KA3, BTAF1. USP3, MYB
Target scan miRwalk	16	CACNA2D1, ATXN7L3B, BTRC, FRYL, RBBP6, VAPB, CD2AP, DCAF7 VEGFA, AA15, EIF4E, SEMA6D, ATXN1L, RAB9A, DCTN5, TMC7
miRmap miRwalk	30	CHPT1, GLT8D2, CAPZA2, EFHD1, CRHBP, RASSF5, AHCYL2, RRAGA, P1LC3B, DAMTSL2, PODXL, CSE1L, WWP1, GLUD1, SMYD4, SMAD2, SNRPA1, AGL, FGF2, CEP85L, TRPC6, ZNF34, ATP6V1G2, CUL2, PRICKLE1, NUP43, ST8SIA6, TTC1, C11H2orf42, GLS2

Supplementary table S4: KEGG pathway analysis of miR-195-5p predicted target genes

KEGG Term	Count	p-value	Genes
bta04151<br>PI3K-Akt signaling pathway	10	0.031	FGF7, YWHAQ, FLT3, MYB, AKT3, PPP2R5C, SGK1, EIF4E, FGF2, VEGFA
bta05132<br>Salmonella infection	9	0.010	DCTN5, MAPK8, TUBA1A, PODXL, CSE1L, AKT3, RAB9A, DYNC1I1, TXN2
bta04010<br>MAPK signaling pathway	9	0.019	RPS6KA3, FGF7, MAPK8, FLT3, CACNA2D1, MKNK1, AKT3, FGF2, VEGFA
bta04150<br>mTOR signaling pathway	8	0.002	RPS6KA3, RRAGA, ATP6V1G2, SEH1L, STRADB, AKT3, SGK1, EIF4E
bta04014<br>Ras signaling pathway	8	0.023	FGF7, MAPK8, FLT3, RASSF5, AKT3, SHOC2, FGF2, VEGFA
bta05207<br>Chemical carcinogenesis - receptor activation	7	0.039	RPS6KA3, FGF7, AKT3, GNAI3, DLL1, FGF2, VEGFA
bta04914<br>Progesterone-mediated oocyte maturation	6	0.004	RPS6KA3, MAPK8, CDC23, AKT3, CDC27, GNAI3
bta04071<br>Sphingolipid signaling pathway	6	0.012	MAPK8, SPTLC1, SMPD1, AKT3, GNAI3, PPP2R5C
bta04114<br>Oocyte meiosis	6	0.013	RPS6KA3, CDC23, YWHAQ, CDC27, PPP2R5C, BTRC
bta04110<br>Cell cycle	6	0.015	SMAD2, WEE1, CDC23, YWHAQ, CHEK1, CDC27
bta04140<br>Autophagy - animal	6	0.026	RRAGA, MAP1LC3B, MAPK8, GABARAPL1, AKT3, WIPI2
bta05017<br>Spinocerebellar ataxia	6	0.027	ATXN2, MAPK8, ATXN1L, AKT3, TBPL1, WIPI2
bta04261<br>Adrenergic signaling in cardiomyocytes	6	0.029	CACNA2D1, AKT3, GNAI3, ATP1B4, PPP2R5C, SCN4B
bta04120<br>Ubiquitin mediated proteolysis	6	0.029	CDC23, CUL2, CDC27, WWP1, UBE4B, BTRC
bta04066<br>HIF-1 signaling pathway	5	0.038	MKNK1, AKT3, CUL2, EIF4E, VEGFA
bta04964<br>Proximal tubule bicarbonate reclamation	3	0.026	GLUD1, GLS2, ATP1B4

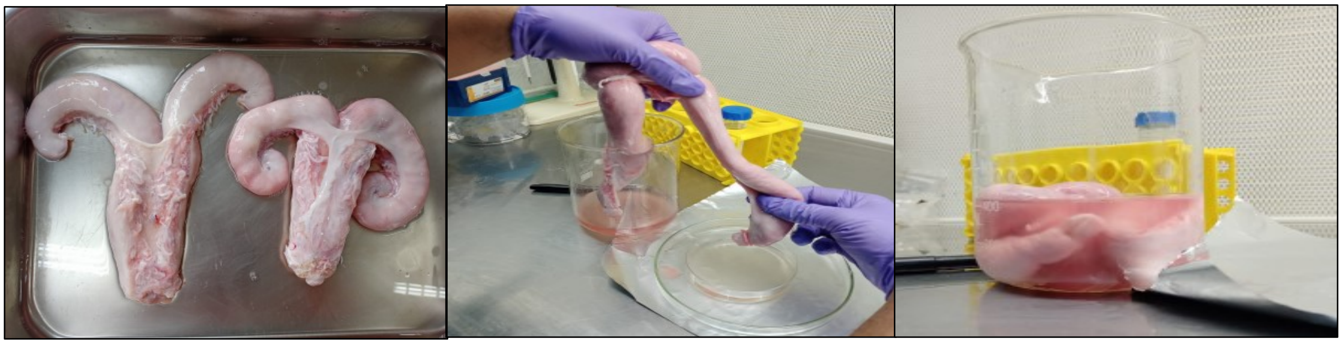

Supplementary figure S1. Isolation of the endometrial epithelial cells

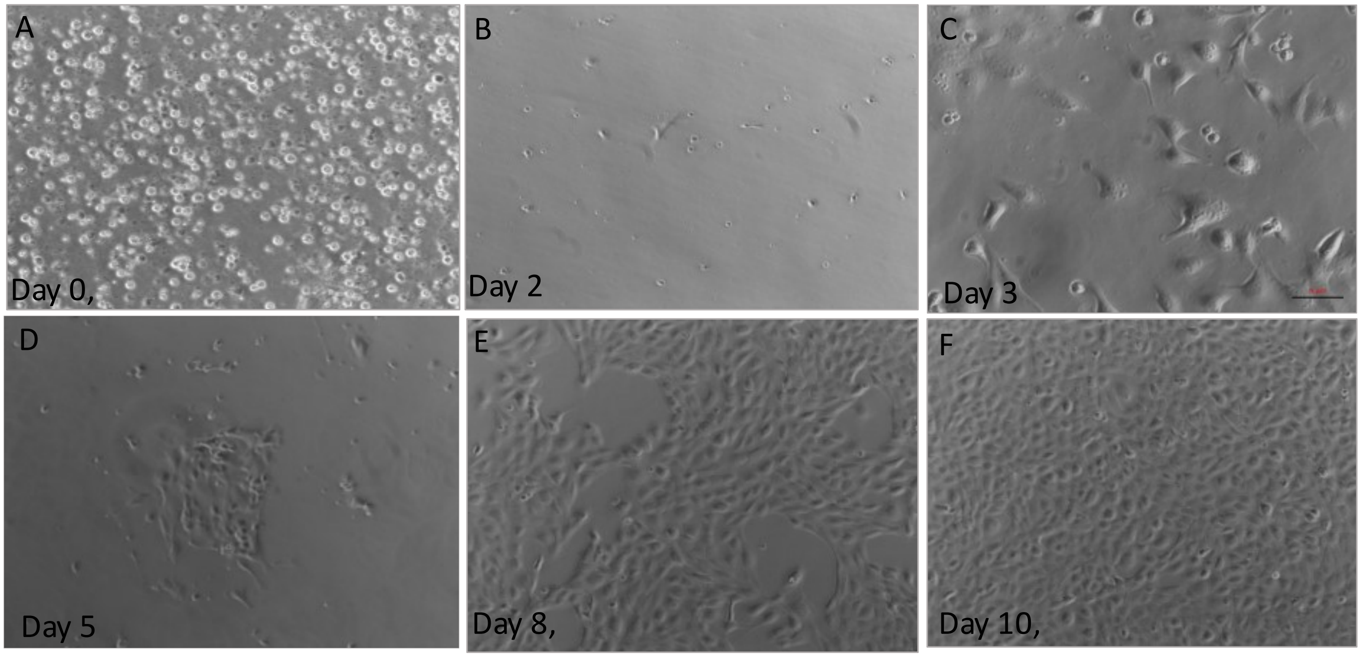

Supplementary Figure S2: Photomicrographs of endometrial epithelial. The morphology of the endometrial epithelial cells was observed with a phase-contrast inverted microscope. (A) epithelial cells were seeded on day0 (B) cells started adhering on day 2 (C) cells started attaining the polygonal shape and dome shaped morphology (D) epithelial cells growing in discrete patches (E) patches of cells had grown and coalesced to form a semi-confluent monolayer of epithelial cells (F) epithelial cells attained the confluency

A. Western blot of TSG101

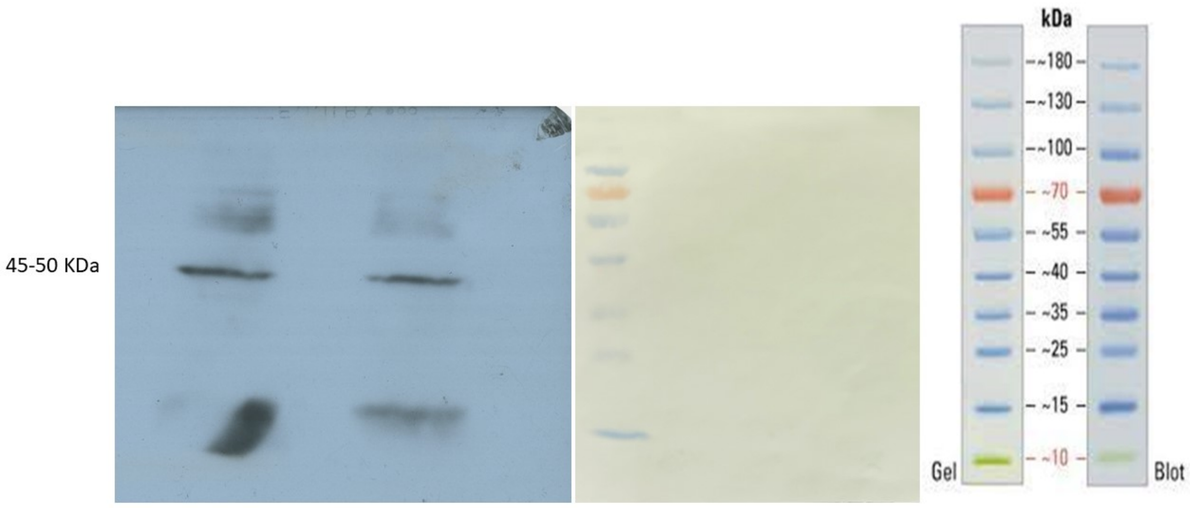

B. Western blot of CD9

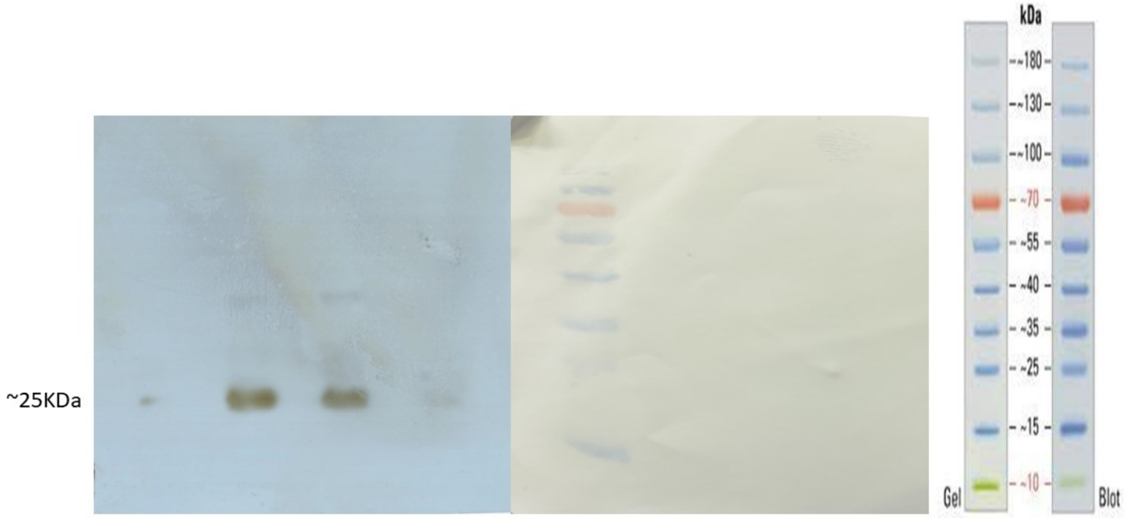

C. Western blot of CD63

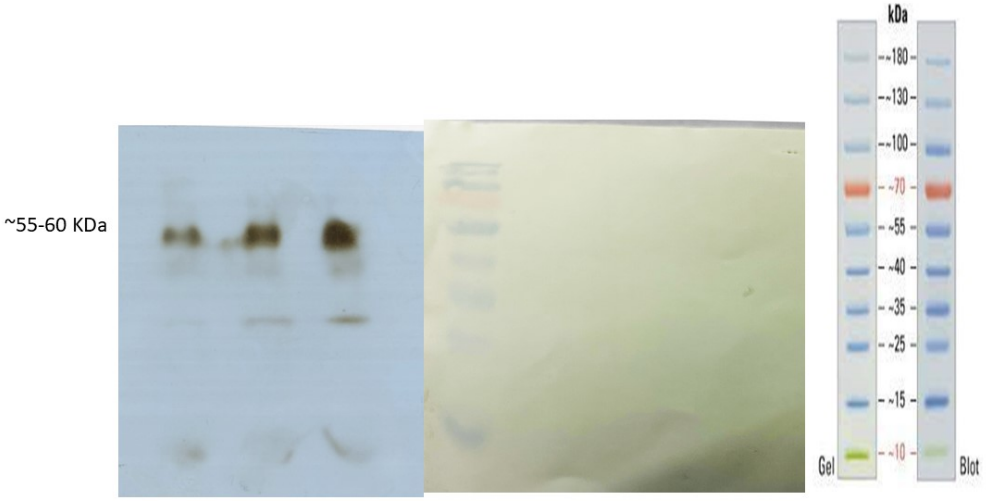

D. Western blot of Calnexin

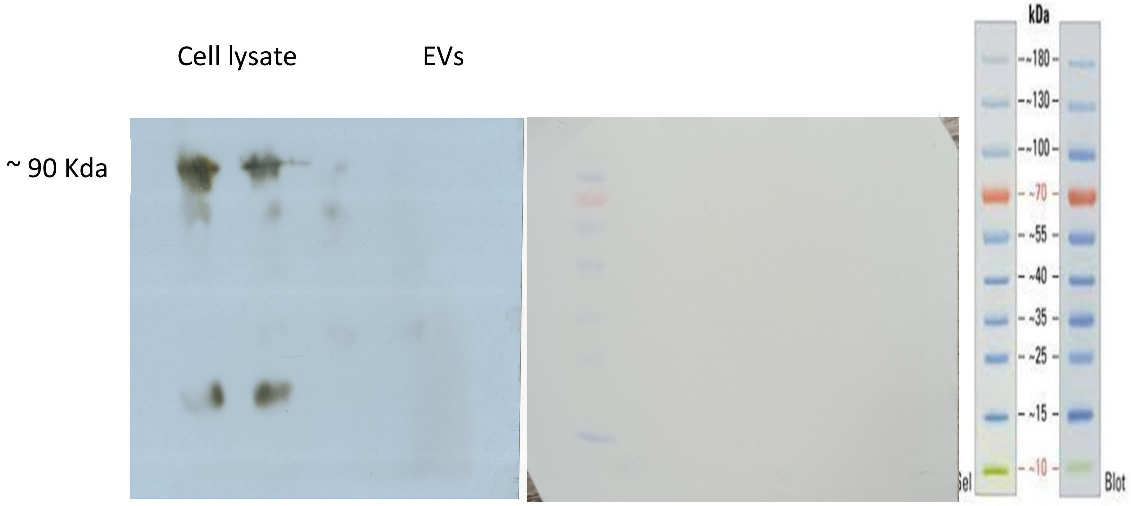

Supplementary Figure S3 (A-D). Western blot images along with ladder for TSG-101, CD-9, CD63, and calnexin antibody. D. CHO cell lysate proteins were used for the positive identification of calnexin while blood plasma derived EVs proteins from Murrah buffaloes for its negative identification.

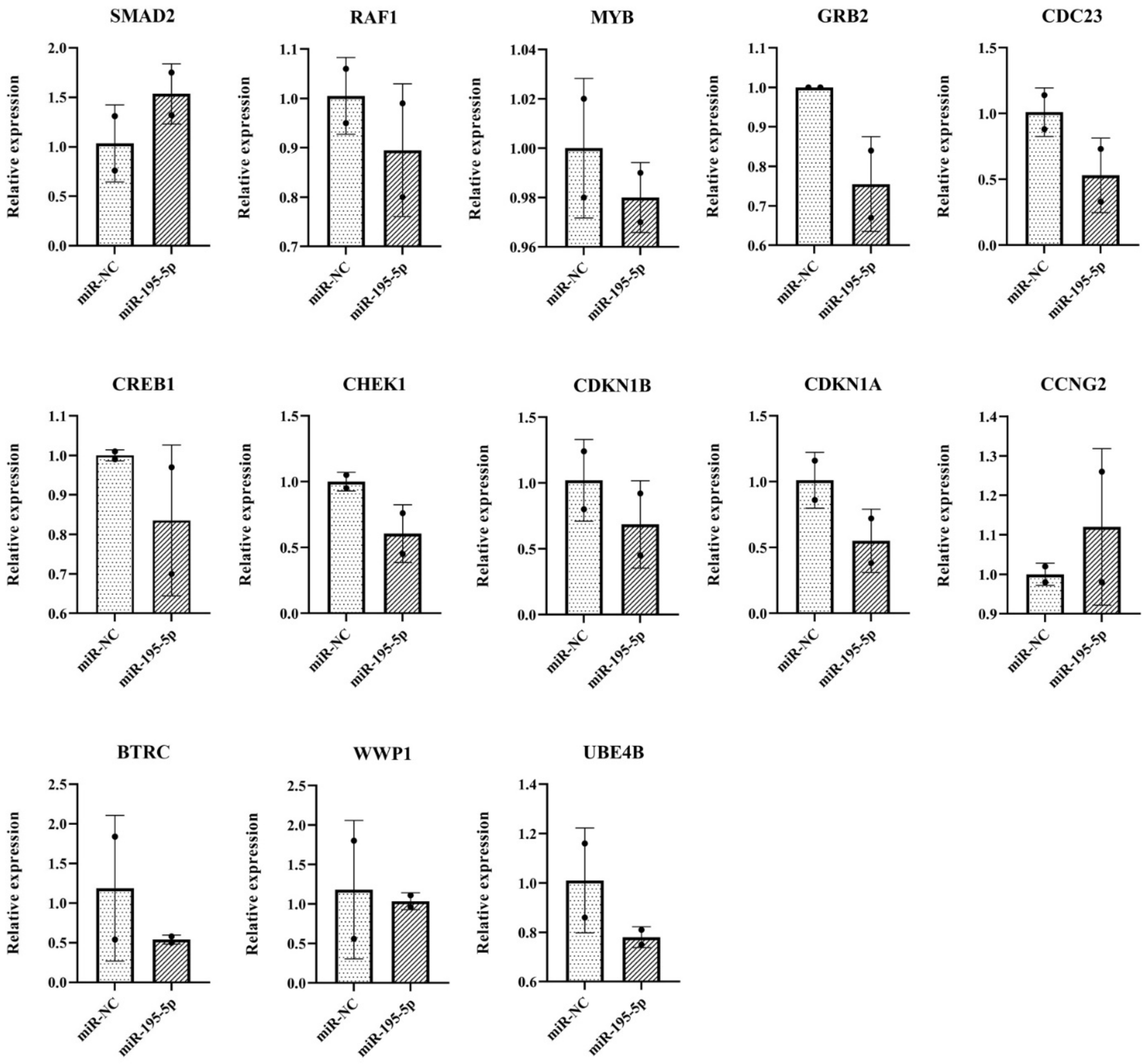

Supplementary figure 4. Relative expression patterns of miR-195-5p target genes (non-significant). The relative expression profiles of the miR-195-5p target genes in the buffalo endometrial epithelial cells transfected with miR-195-5p mimic and miR-NC. Expression values were normalized to GAPDH (n=2). Transfected cells are identified on the X-axis. Y –axis represents relative expression levels.

Supplementary figure 5. PCR-based detection of epithelial cell marker gene cytokeratin (KRT18) and Vimentin in the endometrial primary cells.

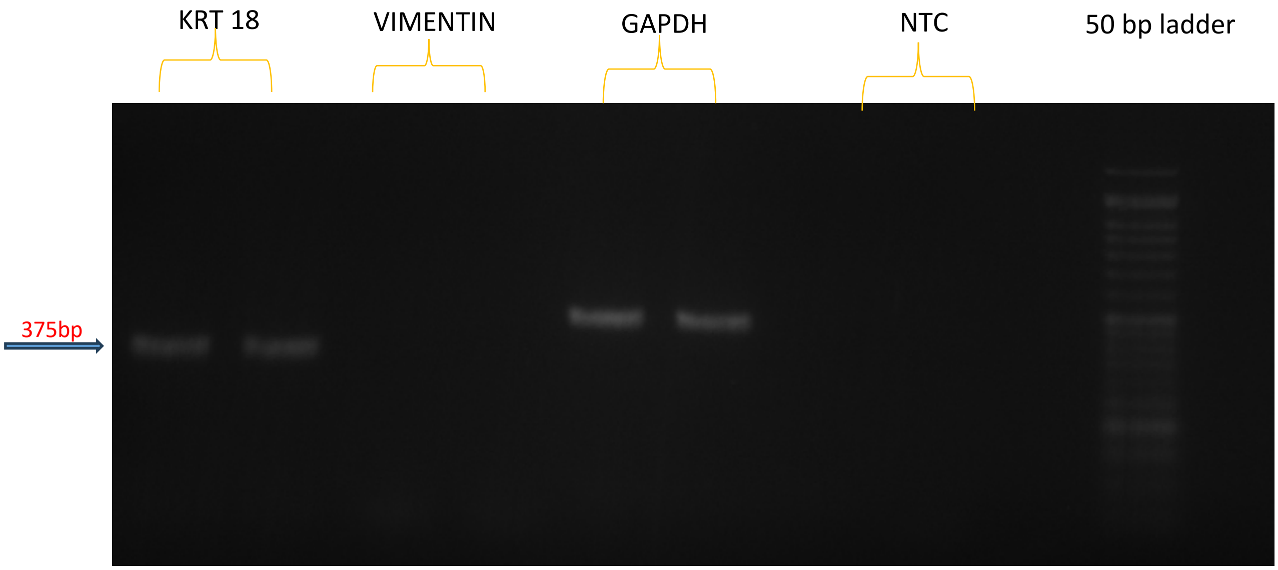

Method 1. Isolation of primary endometrium cells from buffalo uterus

Buffalo uterine epithelial cells were isolated by enzymatic digestion method using 0.25% trypsin–EDTA (T4049; Sigma) as per the protocol described previously with some modifications (Pandey et al., 2020). Briefly, fresh buffalo uteri in the early to mid-luteal phase of the estrous cycle were collected from local abattoirs from apparently healthy, adult buffaloes (n=3). To remove the debris from the uterine horns, each of them was flushed twice with 15-20 mL of warm 1X PBS containing 0.1% bovine serum albumin (BSA) (05482; Sigma) and 100 µg/mL gentamycin (G1272, Sigma). After flushing, the horns were sealed from one side with cotton thread and 10-15 mL of 0.25% trypsin-EDTA was injected into the uterine lumen (Supplementary figure 1). In a beaker containing 1X PBS, uterine horns were maintained for two hours in an incubator at 37°C with 5% CO_2. The suspension of uterine contents was drawn out and passed through a 40 µm cell strainer. The cells adhered to the back of the cell strainer were collected and pooled together after being reverse washed followed by centrifugation at 600 g for 10 minutes. The obtained cell pellet was washed twice with 1X PBS containing 0.1% BSA and subsequently re-suspended in complete media containing high-glucose Dulbecco's Modified Eagle Medium (DMEM) (D5796, Sigma), 10% FBS (Lot# 2106467RP, Gibco) and 1X antibiotic-antimycotic solution (Lot# 2,257,208, Gibco). The cells in the suspension were spun down once more at 600 g for 5 minutes to give it one last washing and then resuspended in a complete culture medium. The cell number and viability were determined by staining the cells with 0.4% trypan blue dye (T8154, Sigma) and viewing it under bright field microscope using a Neubauer's chamber. The cells were seeded at a density of $5x10^4$ cells/cm^2 in a 24 – well plate (Nunc, Thermo Scientific) and incubated at 38.5°C in a 5% CO_2 humidified incubator. Uterine luminal epithelial cells (LE) were collected along with the medium and re-plated into a fresh 24- well plate after being cultured for 24 hours. Up until 70–

80% cell confluency was achieved; the culture medium was replaced every 48 hours. Cells were passaged and then reseeded at a density of $5x10^4$ cells/cm^2 into the next flask.
